# Validation of the group tasks uncertainty model (MITAG) in a German sample

**DOI:** 10.1371/journal.pone.0224485

**Published:** 2019-11-08

**Authors:** Jan-Paul Leuteritz, José Navarro, Rita Berger

**Affiliations:** 1 Ergonomics and Vehicle Interaction, Fraunhofer-Institute for Industrial Engineering (IAO), Stuttgart, Germany; 2 Departamento de Psicología Social y Psicología Cuantitativa, Universitat de Barcelona, Barcelona, Spain; Medical University Innsbruck, AUSTRIA

## Abstract

Task uncertainty is a key factor in teamwork research. This study analyzed the psychometric characteristics of the Spanish Model of Group Tasks Uncertainty (MITAG) in two German samples. The participants (501 team members and 104 team leaders from a German research organization) answered the MITAG together with selected items from the German Job Diagnostic Survey (JDS) and the instrument Ambiguity facets of work (Ambiguitätsfacetten der Arbeit, AfA). Confirmatory factor analysis did not reproduce the original 4-factor structure in the German sample, although the 3 newly identified factors unclarity of goals, new situations, and non-routine resemble the original factors. Results showed sound internal consistency and confirmed the convergent and discriminant validity of the new factors. The MITAG offers a concept-based short scale for researchers and practitioners.

## Introduction

Modern working contexts are increasingly affected by task uncertainty. Unclear objectives, time pressure, and polyvalence are rising across different job types [1]. Research is required to find out how individuals and teams deal effectively with uncertain tasks and this requires measuring task uncertainty.

Task uncertainty is of particular interest in team research, as it may have a direct or indirect (i.e., moderating) influence on team performance. Some have argued that uncertainty was detrimental to performance [2], as adapting to task uncertainty requires the team to spend extra resources on planning and decision-making, thus decreasing efficiency. Others showed that task uncertainty could moderate the relationship between group processes and team performance, such as boundary reinforcement [3] or relational resources among team members [4]. Sicotte and Bourgault [5] identified both, direct effects and moderating effects on team performance. These contradictions largely remain unresolved, since the operationalizations of task uncertainty differ, and since too little attention has been paid to the multidimensionality of this construct. The same contradictions extend to project uncertainty [6], some aspects of which may be closely related to task uncertainty.

Several measurements of task uncertainty were created in the past decades, but they have methodological weaknesses and lack conceptual foundations. For example, Perrow [7] defined task uncertainty by *task variety* (or *number of exceptions*) and *task analyzability*. Other researchers took this work up. Van de Ven and Ferry [8], for instance, created a questionnaire based on the dimensions *task variability* and *difficulty*. Combining measures of difficulty and uncertainty may, however, not be advisable, since task difficulty refers to the worker’s knowledge, skills, and abilities, whereas task uncertainty may instead rather depend on external factors. However, the idea that difficulty is a defining aspect of task uncertainty is still propagated in research, e.g., by Dingsøyr et al. [9], referencing the work by Van de Ven, Delbeq, and Koenig [10]. Withey, Daft, and Cooper [11] compared 12 task uncertainty measurement instruments, targeting *task variety* or *variability*, *difficulty*, *analyzability*, and *predictability* or *insufficient knowledge*. Based on their exploratory factor analysis, they created a 10-item task uncertainty scale, measuring the factors *exceptions* and *analyzability*. However, this instrument has a questionable factor structure; their analyzability scale did not differentiate well between teams and convergent validity was inflated due to shared items [11]. They created the scale based on existing items, instead of a comprehensive theoretical framework of task uncertainty. The validity issues of the instruments analyzed by Withey et al. [11] have affected researchers in need of a short, effective measurement of task uncertainty. Some researchers resolved to pick out small numbers of items from the instruments published by Van de Ven and Delbecq [12] or by Withey et al. [11]. Even though these items apparently reflected different subordinate dimensions of task uncertainty, the factor structure of these reduced instruments was disregarded; global measurements of uncertainty were created by combining items from different subordinate dimensions. This imposes severe limitations, such as those seen in the articles by Nidumolu [13] and by Gardner et al. [4]: results are difficult to interpret when it is not clear what exactly is uncertain about the employees’ work and why. This has implications for the role of uncertainty, for instance as mediator or moderator variable.

Hence, the measurement of task uncertainty is still an unresolved issue. To fill this gap, Navarro, Díez, Gómez, Meneses, and Quijano [14] developed the MITAG, the Spanish Model of Group Tasks Uncertainty (full Spanish name: *Modelo de Incertidumbre de las TAreas del Grupo*). They created a new set of items, taking additional literature on task characteristics into account and providing a more comprehensive conceptual model of task uncertainty that distinguishes better between different dimensions of uncertainty and thus can presumably differentiate better between certain kinds of teams or job types than previous models.

The MITAG pertains to the measurement framework named *Human System Audit* [1] and defines task uncertainty as “the existence of unclear connections or links between what the group must do (work) and the result it will achieve from this work (results)” [1]. Navarro et al. [1] developed it as a synthesis of different task characteristics models. Particularly, the MITAG is based on a review of McGrath’s [15] circumplex model, Campbell’s [16] task complexity model, and the organizational assessment instrument by Van de Ven and Ferry [8], as described by Navarro and colleagues [14]. Instead of the circumplex model, which provides a typology of tasks, the MITAG allows measurement of the different characteristics of a task, which are defined as requirements related to the behaviors required for achieving optimal performance, on a continuum [14]. Compared to the models presented by Campbell or by Van de Ven and Ferry, the MITAG excludes aspects such as complexity, opacity, or difficulty [14], which may be criticized for blending objective and subjective operationalizations.

Navarro et al. [14] defined six initial dimensions of uncertainty and created a set of items, which they validated in a Spanish sample; their exploratory factor analysis resulted in four factors: clarity (Spanish: claridad, six items), diversity (Spanish: diversidad, three items), novelty (Spanish: novedad, six items), and conflict (Spanish: conflicto, three items). Ferràs [17] confirmed this 4-factor model in a second Spanish sample [1]. The factors were defined as follows.

1. “Clarity refers to the knowledge of team members regarding what they must achieve (goals) and how they can achieve them (processes).” [1]. Thus, this factor relates to the success criteria and the possibly unpredictable relationship between a given method and its desired outcome.
2. “Diversity makes reference to the quantity and variety of tasks the work group has to perform” [1].
3. “Novelty refers to those task characteristics that make the group not know which is the best way to perform it and, in addition, that members have to choose among different alternative procedures based on a subjective efficiency criteria.” [1].
4. “Task conflict refers to the possible incompatibilities regarding tasks that are presented to the group, as to whether it is due to discrepancies among different tasks or within one same task, as performing a task efficiently can mean not attending to other tasks the group must also perform”[1].

Navarro et al. [1] reported high cross-loadings between the factors of clarity and conflict, which is understandable as both factors refer to the team’s objectives. Its internal consistency at the lower end of the acceptable range (63. < Cronbach’s α < .68) raised doubts as to whether the MITAG’s dimensions were too abstract to be transferred into another cultural context. Nevertheless, we deemed it important to provide such an instrument in German and thus to validate the MITAG. An English translation of the MITAG is available in the English version of the article published by Navarro et al. [1]. Two bilingual native speakers with a degree in psychology created this English version through a back-translation process [18]; however, it has not yet been validated in an English-speaking sample.

All previous validation studies of the Spanish MITAG were conducted in Spain [14,17]. Compared to Spain, Germany is characterized by a lower power distance [19], which may lead to a different perception of uncertainty and consequently affect the factor structure of the questionnaire. Although the exploration of cultural effects on the MITAG was beyond the aim of this article, we expected that finding sound psychometric results in German samples could contribute to fostering its cross-cultural validity.

Beyond measurements of task characteristics, an alternative approach to assessing uncertainty at work is available: role ambiguity. This construct describes the extent of uncertainty experienced because of missing information with respect to what is expected of a person in the work context. Its supposedly best-known operationalization is the RHL scale, named after its creators Rizzo, House, and Lirtzman [20]. The subordinate dimensions of role ambiguity, named *aspects* or *facets* include uncertainty about responsibilities and criteria for the assessment of the individual’s performance, as well as the uncertainty of not knowing the objectives of the work or the required methods [21]. With respect to the latter, role ambiguity overlaps with the construct of task uncertainty. We concluded that role ambiguity facets that refer to the objectives or methods of the work would be highly related to task uncertainty and that evaluating this relationship would contribute to the theoretical knowledge about the constructs represented in the MITAG. The other facets of role ambiguity are rather focused on social or inter-personal phenomena, which is reflected in their mostly negative correlates to performance or work satisfaction measures, as reported in the literature of the 1980s [21]. These other facets should thus not be interchangeable with task uncertainty.

With respect to other established measures of task characteristics, a measurement of task uncertainty such as the MITAG would not have an overlap but a likely statistical relationship: one can assume that jobs characterized by a high uncertainty regarding work methods or work objectives do not offer sufficient feedback, as operationalized in the Job Diagnostic Survey (JDS). Its German version was created by Schmidt, Kleinbeck, Ottmann, and Seidel [22]. Another differentiated relationship pattern may be expected for worker autonomy: tasks characterized by the MITAG dimensions of clarity and conflict may affect workers with high and low autonomy alike. However, in tasks characterized by high diversity and high novelty, a certain extent of worker autonomy is required: workers need to choose one of many possible methods and autonomously adapt their strategies as they use novel methods.

## Materials and methods

The purpose of the present study was to analyze the psychometric characteristics of the MITAG in two samples of German employees working in a research context. We used confirmatory factor analysis (CFA) to assess the instrument's factor structure. Additionally, we tested internal consistency. We explored the convergent and discriminant validity of the MITAG by analyzing the relationships between its subscales and concepts thought to be associated with uncertainty.

### Participants and data collection

Team members (sample 1) and team leaders (sample 2) from a German research organization completed an online survey (Table 1). Among all invited teams, minimum team size was three members and a leader; mean team size was 6.9 members without counting the leader. Members (sample 1) answered the MITAG questionnaire and the other instruments mentioned below used for evaluating the MITAG’s convergent and discriminant validity; leaders (sample 2) answered only the MITAG items, due to an agreement with the organization to provide a shorter questionnaire for leaders. Mean age was 34.3 years (*SD* = 11.8) in sample 1 and 41.4 years (*SD* = 9.5) in sample 2. Most participants were researchers, while the others worked in the administration, IT departments, public relations (PR), or mechanical workshops. The majority was male.

**Table 1:** Sample description.

	Sample 1: Team members (*N* = 501)		Sample 2: Team Leaders (*N* = 104)	
	*N*	per cent	*N*	per cent
Male participants	343	68.5%	87	83.7%
Female participants	158	31.5%	17	16.3%
Job: researcher	423	84.4%	88	82.2%
Job: other	78	15.6%	19	17.8%

*N* = Number of individuals.

### Ethical standards

Participation was voluntary. By accepting the terms of participation stated at the beginning of the online survey and by finally submitting their data, all participants gave an equivalent of written informed consent, in accordance with the Declaration of Helsinki. The protocol was approved by the workers‘ council of the participating organization.

### Measures

The MITAG consists of 18 items and based on its validation in a Spanish sample [1], the following dimensions emerged: Clarity (Cronbach’s α = .65), Diversity (α = .63), Novelty (α = .68), and Conflict (α = .63). The following list contains examples from the German item set [1]

- Clarity: “We are very clear on what we must achieve with our work” (German: “In meiner Arbeitsgruppe ist es für uns ganz klar was wir mit unserer Arbeit erreichen sollen”)
- Diversity: “There are different ways of doing our job well and we shall select the most efficient one” (German: “In meiner Arbeitsgruppe gibt es unterschiedliche Arten unsere Aufgabe gut zu erledigen und wir sollen dabei die effizienteste auswählen”)
- Novelty: “Frequently, new problems and situations arise, in which we feel confused about the best way of working” (German: “In meiner Arbeitsgruppe treten oft neue Probleme und Situationen auf, bei denen wir nicht wissen wie wir sie am besten erledigen”)
- Conflict: “From time to time, doing one task well requires us to neglect another task” (German: “In meiner Arbeitsgruppe müssen wir um eine Aufgabe gut zu erledigen immer wieder andere Aufgaben vernachlässigen”)

To analyze the convergent and discriminant validity of the MITAG’s factors, we prioritized well-established instruments in German with peer-reviewed validation studies. We measured:

1. the factor *work method ambiguity* (WMA, German: *Klarheit über die Arbeitsmethoden)* in the instrument *Ambiguity facets of work* (German: *Ambiguitätsfacetten der Arbeit*, AfA) by Schmidt and Hollmann [21], consisting of three items (α = .91); and
2. the dimensions *autonomy* (α = .76) and *feedback on the job* (α = .87) from the German version of the JDS, each dimension represented by three items [22].

### Procedure

Based on the recommendation by the ITC, we applied a back-translation method to ensure cultural and linguistic differences were taken into account [23] when translating the MITAG to German. The following translators were involved:

• Translator A (female German native speaker, fluent in Spanish (C1), organizational psychologist)

• Translator B (male Spanish native speaker, fluent in German (C1), general psychologist)

• Translator C (male German native speaker, fluent in Spanish (B2), organizational psychologist)

• Translator D (female German and Spanish bilingually raised native speaker, Spanish language teacher, no access to source text)

We followed the steps proposed by [24], as far as applicable; during the process, we applied the recommendations by the ITC [23]:

1. Forward-translation of the MITAG from Spanish to German by translators A and B.

2. Revision of the German versions by 3 employees of German companies, in order to identify the items they found easiest to understand.

3. Harmonization of the translations and the input by the employees, by translator C.

4. Back-translation of the harmonized translation to Spanish by translator D.

5. The back-translation was reviewed by translator C, in cooperation with B.

6. Back-translation review by Translators A and C.

7. Adaptation of the German translation, based on the discrepancies or possible shifts in meaning or context, as identified through the back-translation.

8. Second back-translation of the new version by translator D.

9. Back-translation review by translators A and C, who now agreed that sufficient conversion had been achieved.

We did not use the English version as a second input, as it had not yet undergone thorough validation based on empirical data. As in the original version, we combined the items with a 5-point Likert-scale.

To collect the data, we first obtained permissions from the organization’s Human Resources director and by the directors of several divisions. Then, we identified the teams of sufficient size based on organization charts, and sent personalized access codes to the online survey via encrypted email. As an incentive, participants who returned a completed questionnaire could participate in a lottery. For reasons of data protection, we used two data files per sample: one containing the answer data and a participant code, and another file to associate the participants’ real names and addresses to their participant codes. All files were stored on encrypted password-protected virtual drives, and the file containing real names was only available to one researcher. The workers’ committee of the organization checked for ethics and data privacy issues and approved the study.

We used AMOS version 22 for the CFA. We chose χ^2^, χ^2^/*df* ratio, Root Mean Square Error of Approximation (RMSEA: [25]) and TLI (Tucker-Lewis-Index: [26]) for evaluating SEM fit. We preferred TLI over CFI (Comparative Fit Index: [27]) as it is more conservative [28]. To calculate Composite Reliability (CR), Average Variance Extracted (AVE), Average Shared Variance (ASV) and Maximum Shared Variance (MSV) we used a tool by Gaskin [29] and we chose Cronbach’s α to assess internal consistency. We used the sample of leaders (sample 2) for exploratory factor analysis and reliability assessment and we based all other analyses on the data from the members (sample 1).

## Results

### Factor structure

With respect to CFA, we set the cut-off-values at 0.95 for TLI and at 0.6 for RMSEA [30]. We accepted χ^2^/*df* ratio below 5 [31]. We used χ^2^ only for model comparisons, since it becomes significant in larger samples, even when model fit is acceptable [32].

In the CFA, the factor structure identified by Navarro et al. [1] in a Spanish sample, containing the four dimensions of clarity, diversity, novelty, and conflict, could not be reproduced in the German sample 1 (Table 2). Neither fitted a model based on the original six theoretical dimensions [14], from which these four factors had emerged. We refrained from testing one- or two-factor models, for the following reasons: (1) Since the theoretical model does not support such a model, and since we wanted to measure task uncertainty as a multidimensional construct, and (2), since the fit problems seemed to arise from the fact that the items that were supposed to represent one factor did not show enough common variance.

**Table 2:** Model fit parameters.

Model	Sample	χ^2^	*df*	χ^2^/*df*	*p* (χ^2^)	TLI	RMSEA
Original	Members	410.12	118	3.48	.00	0.82	0.07
Original	Leaders	213.84	118	1.81	.00	0.71	0.09
New	Members	53.00	22	2.41	.00	0.95	0.05

χ^2^ is the Chi-Square statistic, and *df* is the respective number of degrees of freedom. *p* (χ^2^) is the significance level of the χ^2^ statistic. TLI = Tucker-Lewis Index; RMSEA = Root Mean Square Error of Approximation [33].

We therefore performed an exploratory Oblimin-rotated principal axis analysis on sample 2 (leaders). The requirements to perform the EFA were met: the Kaiser-Meyer-Olkin Index, which had to be greater than .50, was .69, and Bartlett’s test of sphericity was significant as required (χ^2^(153) = 542.29, *p* = .00). Six factors with an eigenvalue above 1 were identified (Table 3). However, many items showed high cross-loadings. We deleted nine items and preserved three factors. A new CFA in sample 1 confirmed the new model. The differences in χ^2^ between the 4-factor model and the new model (Table 2) justified accepting the latter. The new factors are:

- Factor 1: *unclarity of goals*. Items 3, 15, and 18 either refer to a lack of definition or a conflict between goals, or to a very general idea of what the group is expected to achieve in the long run. If a person scored high on factor 1, one would assume that the team leader had failed to set team goals well.
- Factor 2: *new situations*. Items 5, 8, and 14 refer to short-term demands or situational changes that produce uncertainty or conflict concerning the chosen method or prioritized objective. High scores on factor 2 would presumably result from the organizational environment rather than from within the team, contrary to factor 1.
- Factor 3: *non-routine*. Items 6, 13, and 17 relate to automated and routine work, or to monotonous demands and simple information. Therefore, high scores on this factor represent a lack of standardization, predictability, or routine of the task contents and procedures.

**Table 3:** Principal axis factoring–structure matrix.

	*M* (*SD*)	1	2	3	4	5	6
Item 3	2.01 (0.68)	.42	-.01	.09	.11	**.72**	.01
Item 15	2.36 (0.83)	.39	-.05	.18	.08	**.85**	.02
Item 18	2.81 (0.86)	.38	-.11	.28	-.02	**.66**	-.11
Item 5	3.26 (0.94)	**.65**	-.05	.00	.04	.33	-.25
Item 8	3.49 (0.81)	**.64**	.23	.16	.31	.26	-.14
Item 14	3.13 (0.69)	**.66**	.17	.21	.13	.31	.13
Item 6	4.53 (0.57)	.21	.11	**.60**	.04	.02	.18
Item 13	4.37 (0.70)	.15	.10	**.53**	.18	-.09	-.41
Item 17	3.82 (0.87)	-.02	.15	**.65**	.16	.15	-.15

Items were recoded according to instructions. Surviving items only. Sample 2 (*N* = 104 leaders).

### Characteristics of the new model

#### Internal consistency

We calculated Cronbach’s α in both samples. It was highest at .77 and lowest at .58 (see Table 4), which corresponds to the findings reported by Navarro et al. [14].

**Table 4:** Convergent and discriminant validity.

	Mean_1_	SD_1_	α_1_	α_2_	CR_1_	AVE_1_	MSV_1_	ASV_1_
MITAG—Unclarity of goals	2.33	0.67	.77	.80	.79	.55	.37	.25
MITAG—New situations	3.22	0.70	.68	.68	.68	.41	.37	.21
MITAG—Non-routine	4.04	0.58	.64	.58	.63	.36	.13	.08

α is Cronbach’s α. CR is Composite Reliability. AVE is Average Variance Extracted. MSV is Maximum Shared Variance. ASV is Average Shared Variance. Index 1 indicates data from sample 1 (members, *N* = 501), index 2 refers to data from sample 2 (leaders, *N* = 104). The analysis was based on sample 1; however, α-values from sample 2 were included to demonstrate their variability, particularly their lower bounds.

#### Convergent and discriminant validity of the MITAG

We calculated CR, AVE, ASV and MSV in sample 1. We accepted convergent validity at item level with CR > .7 and AVE > .5; for discriminant validity at item level, we required MSV < AVE, ASV < AVE, and the square root of AVE to be greater than the inter-factor correlations [34]. Convergent validity of unclarity of goals was satisfying, while the other two factors did not meet the quality criteria: CR and AVE were below the thresholds for new situations, and non-routine. Discriminant validity of the MITAG instrument was fully confirmed (Table 4).

Unclarity of goals correlated highest with AfA’s WMA, which particularly represents work ambiguity or uncertainty. It was also moderately related to the JDS measure feedback on the job, a specific work characteristic that induces a certain type of ambiguity. Unclarity of goals was less associated with autonomy, a measure less likely to show such a direct relationship to task uncertainty. This pattern repeated itself with smaller correlation coefficients for the MITAG factor of new situations: it correlated moderately with AfA’s WMA, lower with JDS’s feedback on the job, and insignificantly with JDS’s autonomy. Non-routine showed a higher correlation with autonomy than with feedback on the job or with the WMA. This is plausible, as one can expect non-routine tasks to require more autonomy of the individual worker. The MITAG dimensions were reverse coded compared to all other measures used, which accounts for nearly all correlations being negative (Table 5).

**Table 5:** Pearson correlations among MITAG and selected criteria in sample 1.

		1	2	3	4	5	6
1	MITAG–unclarity of goals	(.78)					
2	MITAG–new situations	.47**	(.68)				
3	MITAG–non-routine	.22**	.21**	(.64)			
4	AFA–work method ambiguity	-.59**	-.39**	-.09*	(.91)		
5	JDS–autonomy	-.12**	-.09	.23**	.22**	(.76)	
6	JDS–feedback on the job	-.30**	-.14**	.10*	.38**	.38**	(.87)

** indicates significance at *p* < .01.

* indicates significance at *p* < .05. The main diagonal (in brackets) contains Cronbach’s α. *N* = 501 team members.

#### Measurement invariance

We tested for configural measurement invariance, which refers to the same factor structure applying to the respective subgroups, and for metric invariance, which represents equality of factor loadings. We accepted metric invariance if ΔCFI was .01 or smaller [35]. The MITAG showed configural invariance with respect to job type and gender. ΔCFI was acceptable at .008 when testing for metric invariance between researchers and the group of other job types (administration, IT, PR, and workshops). However, the factor loadings differed between men and women (ΔCFI = .035).

#### Criterion-based validity

As an external validation criterion, in sample 1 we tested whether the MITAG distinguished between researchers and administration staff. We expected the latter to score lower on all task uncertainty dimensions, since we assumed their work to be more routine-based and predictable, and their objectives to be better defined compared to researchers. We set the Type I error at α = .05 and checked for normal distribution using the KS test and for homoscedasticity using Levene’s test. As the respective preconditions were met, we applied *t*-tests with Bonferroni corrected significance levels for the three outcome variables. Indeed, compared to their colleagues in administration, researchers scored higher on unclarity of goals (*t*(463) = 5.22, *p* < .017), new situations (*t*(463) = 3.00, *p* < .017), and non-routine (*t*(463) = 7.48, *p* < .017).

## Discussion

This study contributed to the state of the art by providing a new instrument for measuring task uncertainty in German-speaking samples and by advancing our understanding of cultural factors that influence the measurement of this multifaceted construct. Until today, common instruments for the measurement of task uncertainty either lacked a sound theoretical framework, for example when combining the incompatible constructs of difficulty and uncertainty, or they had methodological weaknesses, such as problematic factor structures or validity problems. With this work, a shortened version of the MITAG became available for German-speaking samples. It distinguishes between different job types and measures three factors of task uncertainty, while avoiding the problematic dimension of difficulty. It features an elaborated conceptual framework [14] and thus allows interpretation of results in a greater theoretical context.

The new factor structure furthers our understanding of how task uncertainty reflects the cultural or organizational context in which it is measured. To our knowledge, the relevance identified here of the source of uncertainty–in contrast to the type of uncertainty–has not yet been taken into account in any other relevant instrument.

### Main findings

The main finding is the new factor structure, composed of the three dimensions: unclarity of goals, new situations, and non-routine. The first factor, unclarity of goals, joins items that refer to the extent to which the team leader has failed to define general or long-term goals or objectives. The second factor, new situations, refers to the uncertainty produced by conflicting or fast-changing short-term demands from outside the team. Non-routine is the extent to which processes, methods and input information are standardized or well known to the team members.

One may argue that even though the original four-factor model did not meet our previously defined quality criteria, it still had mediocre fit and could have been maintained. However, in this case we would still have had to analyze why model fit was worse in the German sample, or why certain items behaved differently than which was expected. Discarding the old structure and looking for a new one helped us make assumptions on the reasons for this and identify improvement potentials. Even without these improvements, the shortened MITAG may already be used in German samples. Since the MITAG has not yet been validated in any other culture, we could only draw conclusions from the original validation study and our own data presented here. We ruled out issues with the translation as a possible cause of the issues with the factor structure, since the items were phrased in common, non-expert language, and since we had applied a back-translation process. Further down, we propose two other explanations: (1) Cultural differences in answering patterns, and (2) double meanings in items, which create additional room for cultural differences to be reflected in the answers.

The factor structure identified in both German samples is, indeed, not as different from the original 4-factor structure as it may appear. Navarro et al. [14] also reported cross-loadings between the clarity dimensions and the conflict dimension; in our German samples, such items were joined to the new factor of unclarity of goals. Nevertheless, the factor of new situations indicates a possible cultural difference: while Spanish participants seemed to base their answers rather on the type of uncertainty experienced, German participants appeared to focus on the source of the uncertainty. This may have been due to the difference in power distance between the Spanish and German samples. With a lower power distance in Germany [19], employees may be more inclined to demand good leadership, including well-defined objectives. They apparently reflect more openly on who is responsible for their uncertainty. While, initially, the items had been created solely to distinguish types of uncertainty, some of them also reflect the source of uncertainty. Possibly, these two dimensions of item similarity influenced the inter-item correlations simultaneously. Under such circumstances, the items are unlikely to be parallel or interchangeable. Thus, we expect this effect to have caused the issues with the convergent validity of the factors of new situations and non-routine, regarding CR and AVE.

### Limitations

The first limitation of the present study refers to the composition of the samples. They contained many more men than women, which is relevant since the MITAG failed to prove gender-invariant. The overrepresentation of researchers, compared to other job types, was uncritical, due to the demonstrated measurement invariance. Furthermore, some teams from the category of other job types scored higher than expected on the MITAG. However, this may not be an issue of the instrument itself but of the selected samples. Furthermore, compared to the original study, our sample was composed differently. The original study included employees from a hotel, from a public administration, as well as students of psychology; our sample included only employees of an R&D organization. However, as the MITAG showed metric invariance between job types in our sample, it appears that culture rather than job type made the difference regarding the factor structure. Future studies should collect data from samples with a better gender balance, and data should include a large sample of employees working in jobs in which low task uncertainty can be more safely assumed, such as product assembly or other highly structured work.

Second, the sampling procedure may have led to unknown self-selection effects among participants, thus introducing bias into the scores. However, the MITAG differentiated well between job types, which means there was not, at least, any ceiling effect caused by self-selection.

As argued above, the results suggest that the factor structure of the MITAG may depend on the cultural or organizational context. We collected data from an organization highly engaged in knowledge work and innovation. This may even have had an impact on employees in jobs we assumed to be characterized by lower task uncertainty, such as administration. This is yet another reason for collecting more data from a larger variety of jobs, particularly with presumably lower task uncertainty, and thus enhancing the evidence base.

Another limitation is that the resulting German version of the MITAG now only has limited comparability to the Spanish original. Even though the key aspects of the original instrument were preserved, this study is considered a first step towards creating a new version that would hopefully be applicable in both cultures–and thus be more comparable. One main advantage of the MITAG is the measurement of subordinate dimensions of task uncertainty–and creating an instrument with an interculturally stable factor structure would be a great achievement.

For future research, we further recommend adapting the MITAG questionnaire to resolve the identified validity issues, and to create an instrument with a factor structure that holds in different national cultures. This could possibly be achieved by rephrasing the items that we deleted for not fitting into the new factor structure. In our opinion, items 2, 7, 10, 11, and 12 should reflect the distinction between source and type of uncertainty better than they do now. Items 1 and 7 should not mention team objectives anymore, to emphasize what they are actually about: diverse requirements. Item 4 could be rephrased to address the construct of novelty without mentioning the topic of work autonomy. These changes might result in a new instrument with a factor structure that is applicable across cultures. We recommend testing such an adapted version in another sample, or even another culture. Despite the specified limitations and the recommendation to rephrase and retest some of the items, the results support the use of the MITAG in German-speaking samples, following the approach presented here and using the new factor structure.

### Theoretical and practical implications

The results presented above show that measurements of uncertainty likely depend on cultural factors. This finding is relevant for researchers working with measurements of uncertainty, particularly if these measurements are used across cultures. For practitioners who measure task uncertainty in the context of organizational evaluations or interventions, it is an important finding that task uncertainty is, in any case, a multidimensional construct and that different subordinate factors may play different roles. The instrument that resulted from this validation study is short and practical for use in German samples and has a solid theoretical foundation.

## Conclusions

The MITAG showed a different factor structure in the German samples from the one obtained from Spanish samples. The German translation produced a sound factor structure and evidence of validity in the two given samples. However, it may still be improvable. For future research, we recommend adapting the MITAG questionnaire to increase its convergent validity, and to create an instrument with a factor structure that holds in both national cultures. Additionally, the results indicate that Germans tend to distinguish the source of uncertainty rather than by what is uncertain about the task.

## Supporting information

S1 Appendix Item table. The file contains the items of the MITAG in English, German, and Spanish language.
(DOCX)

S1 File Dataset of sample 1 in SPSS format. The file contains the above-mentioned anonymized data provided by team members.
(SAV)

S2 File Dataset of sample 2 in SPSS format. The file contains the above-mentioned anonymized data provided by team leaders.
(SAV)
