# Supplementary material for: Validation of the group tasks uncertainty model (MITAG) in a German sample
Source: PLoS One. 2019 Nov 8;14(11):e0224485. doi: 10.1371/journal.pone.0224485 (PMC6839861; doi:10.1371/journal.pone.0224485)
Supplement: S1 Appendix — The file contains the items of the MITAG in English, German, and Spanish language. (DOCX) [file pone.0224485.s001.docx]

**S1 Appendix. Item table.**

MITAG – Model of Group Tasks Uncertainty (Modelo de Incertidumbre de las TAreas del Grupo)

| Item number.  (see notes). | English | Spanish (original) | German |
| --- | --- | --- | --- |
|  |  | „En mi grupo de trabajo:“ | „In meiner Arbeitsgruppe…“ |
| 1 (Cl) | To achieve our goals, we need to handle a lot of different information. | Para lograr nuestros objetivos necesitamos manejar mucha información distinta. | müssen wir, um unsere Ziele zu erreichen, mit vielen unterschiedlichen Informationen umgehen können. |
| 2 (i) (Co) | In general, the different objectives demanded are usually compatible. | Por lo general, los distintos objetivos que nos piden suelen ser compatibles. | sind die einzelnen Ziele, die wir erreichen sollen, im Allgemeinen miteinander im Einklang. |
| 3 (Cl) | We are very clear on what we must achieve with our work. | Tenemos muy claro qué es aquello que debemos conseguir con nuestro trabajo. | ist es für uns ganz klar was wir mit unserer Arbeit erreichen sollen. |
| 4 (No)) | There are several ways of doing our task well  and we must choose the most efficient one. | Hay diversas formas de hacer bien nuestra tarea y debemos elegir la más eficiente. | gibt es unterschiedliche Arten unsere Aufgabe gut zu erledigen und wir sollen dabei die effizienteste auswählen. |
| 5 (i) (Cl) | We frequently encounter new problems and situations in which we feel confused about the best way to work. | Con frecuencia surgen nuevos problemas y situaciones en las que nos sentimos confusos sobre la mejor forma de trabajar. | treten oft neue Probleme und Situationen auf, bei denen wir nicht wissen wie wir sie am besten erledigen. |
| 6 (i) (No) | Our task is performed automatically and almost without thinking. | Nuestra tarea se realiza de forma automática y casi sin pensar. | kann unsere Aufgabe automatisch, fast ohne nachzudenken, erledigt werden. |
| 7 (Di) | We have very diverse tasks that force us to manage multiple situations. | Tenemos tareas muy diversas que nos obligan a gestionar informaciones múltiples. | haben wir sehr unterschiedliche Ziele, die uns zwingen vielfältige Informationen zu managen. |
| 8 (Co) | We often receive demands that are difficult to attend to at the same time. | A menudo recibimos demandas difíciles de atender al mismo tiempo. | erhalten wir immer wieder Anforderungen, die wir nur schwer zu gleichen Zeit erfüllen können. |
| 9 (i) (Cl) | It becomes confusing to know what we must achieve with our work. | Nos resulta confuso saber qué es aquello que debemos conseguir con nuestro trabajo. | ist es für uns unklar was genau wir mit unserer Arbeit erreichen sollen. |
| 10 (i) () | There is only one way of doing our work well. | Solo hay una manera de hacer bien nuestra tarea. | gibt es nur eine Art unsere Arbeit am besten zu erledigen. |
| 11 (Cl) | There are few doubts regarding how to do our job well. | Existen pocas dudas en torno a la manera de hacer bien nuestro trabajo. | gibt es nahezu keine Zweifel darüber, wie wir unsere Arbeit am besten zu erledigen ist. |
| 12 | Our job continuously poses new situations to which we must respond. | Nuestro trabajo continuamente nos plantea situaciones nuevas a las que debemos responder. | führt unsere Arbeit ständig zu neuen Situationen auf die wir antworten müssen. |
| 13 (i) | We attend few demands that are different and use simple information. | Atendemos pocas demandas diferentes y utilizamos información sencilla. | erfüllen wir nur wenige unterschiedliche Anforderungen und benutzen wir einfache Informationen. |
| 14 | Doing a task well often requires not attending to others. | Hacer bien una tarea a menudo nos exige desatender otras. | müssen wir um eine Aufgabe gut zu erledigen immer wieder andere Aufgaben vernachlässigen. |
| 15 (Cl) | We have clear and well-defined objectives. | Tenemos unos objetivos claros y bien definidos. | haben wir klar und eindeutig festgelegte Ziele. |
| 16 | Part of our task consists of deciding, from among the possible ways of doing it, which is the best in each case. | Parte de nuestra tarea consiste en decidir, de entre varias maneras posibles de hacerla, cuál es la mejor en cada caso. | besteht ein Teil unserer Aufgabe darin aus verschiedenen Arten diese zu erledigen die beste auszuwählen. |
| 17 (i) | Our job is monotonous. | Nuestro trabajo es rutinario. | erledigen wir Routinetätigkeiten. |
| 18 (Cl) | There is clear knowledge regarding the processes required to achieve our goals. | Existen conocimientos claros sobre los procesos requeridos para conseguir nuestros objetivos. | gibt es genaue Kenntnisse über die für unsere Zielerreichung notwendigen Prozesse. |

Notes. (i) inverse coded item; (Cl) Clarity; (No) Novelty; (Di) Diversity; (Co) Conflict.
